# Patients’ Readiness to Exercise After Cardiac Surgery: A Single-Center Observational Study

**DOI:** 10.3390/healthcare14132012

**Published:** 2026-07-06

**Authors:** Eleni Anagnostopoulou, Konstantinos Giakoumidakis, Anastasia A. Chatziefstratiou, Nikolaos V. Fotos, Athina Patelarou, Evridiki Patelarou, Hero Brokalaki

**Affiliations:** 1 Department of Nursing, School of Health Sciences, National and Kapodistrian University of Athens, 11527 Athens, Greece; anele293@yahoo.com (E.A.); nikfotos@nurs.uoa.gr (N.V.F.); heropan@nurs.uoa.gr (H.B.); 2 Department of Nursing, School of Health Sciences, Hellenic Mediterranean University, 71410 Heraklion, Greece; apatelarou@hmu.gr (A.P.); epatelarou@hmu.gr (E.P.); 3 Laboratory of Evidence-Based Healthcare, Education and Clinical Protocols, Department of Nursing, School of Health Sciences, Hellenic Mediterranean University, 71410 Heraklion, Greece; 4 Cardiac Surgery Unit, General Pediatric Hospital of Athens “Agia Sophia”, 11527 Athens, Greece; a.chatziefstratiou@yahoo.gr

**Keywords:** cardiac rehabilitation, exercise, cardiac surgery, readiness

## Abstract

**Highlights:**

- Patients with a moderate level of preoperative physical activity were more likely to be ready for exercise three months after cardiac surgery.
- Patients with a high level of preoperative physical activity demonstrated greater readiness for postoperative exercise.
- The sex is the major factor affecting patients’ readiness to exercise.

**What are the main findings?**

- Patients with a moderate level of preoperative physical activity were more likely to be ready for exercise than those with a low level of activity.
- Patients with a high level of preoperative physical activity demonstrated greater readiness for postoperative exercise.

**What are the implications of the main findings?**

- The findings of this study may enhance healthcare professionals’ ability to assess patients’ functional status and readiness for exercise following cardiac surgery, facilitating the early identification of mobility limitations and rehabilitation needs.
- The incorporation of functional status assessments into routine clinical practice may support individualized postoperative care planning, promote early mobilization, and contribute to improved recovery outcomes after cardiac surgery.

**Abstract:**

**Background/Objectives**: The prolonged bed rest of patients after cardiac thoracic surgery is associated with many deteriorations in their lives. This study examined patients’ readiness to exercise after cardiac surgery in Greece. **Methods**: This study employs a cross-sectional observational design. Data collection occurred from April to August 2024, and 98 patients who underwent cardiac surgery were enrolled in the study. Patient readiness was assessed via the Greek version of the “Readiness to Change Exercise Questionnaire,” which contains 13 questions divided into four subscales: pre-contemplation, contemplation, preparation, and action. Demographic and clinical data were obtained from medical records, while readiness for exercise was assessed by telephone interview three months after surgery. Data analysis was performed using IBM SPSS 21.0. **Results**: The mean age (standard deviation) was 64.2 (±10.7 years), while the majority were men (72.4%). In the “pre-contemplation”, “contemplation”, “preparation”, and “action” subscales, 11.2%, 11.2%, 24.5%, and 53.1% of the patients were allocated, respectively. Male patients (OR = 3.3, 95% CI 1.0–10.4, *p* = 0.042) were more likely to be ready for exercise than female patients. Compared with patients with low levels of preoperative physical activity, those with moderate and high levels of physical activity were more likely to be ready for exercise (OR = 5.2, 95% CI 1.4–18.7, *p* = 0.012 and OR = 7.4, 95% CI 2.1–26.0, *p* = 0.002, respectively). **Conclusions**: About half of the patients who have undergone cardiac surgery appear to have a good intention of exercising. Parameters such as sex, marital status, and preoperative exercise levels were significantly associated with these patients’ readiness to exercise.

## 1. Introduction

The Transtheoretical Model (TM) was first introduced by Prochaska and DiClemente in 1983, and proposes that behavior change occurs through a series of six stages: pre-contemplation, contemplation, preparation, action, maintenance, and termination [1,2]. The model has been widely applied to the study of various health-related behaviors, including smoking cessation, weight management, physical activity, and condom use [1]. Marshall and Biddle highlighted the need for further research to better define the characteristics of each stage and to clarify behavioral responses and transitions between stages of change [3].

Recent evidence further highlights the importance of cardiac rehabilitation (CR) in improving cardiovascular outcomes. Brown et al. published an updated scientific statement from the American Heart Association and the American Association of Cardiovascular and Pulmonary Rehabilitation regarding the core components of cardiac rehabilitation programs [4]. The authors reviewed the scientific evidence accumulated since the previous update in 2007 and highlighted the evolving role of cardiac rehabilitation in the secondary prevention of cardiovascular disease. The updated framework emphasizes comprehensive patient assessment, nutritional counseling, weight management, cardiovascular risk-factor control, psychosocial management, aerobic and resistance exercise training, and physical activity counseling. In addition, the statement introduces program quality assessment as a new core component and recognizes the increasing role of home-based, hybrid, and telehealth rehabilitation models. The authors concluded that high-quality cardiac rehabilitation programs improve functional capacity, quality of life, and cardiovascular outcomes, while addressing the persistent challenges of low participation rates, poor adherence, and inequalities in access to rehabilitation services [4].

The TM has broad applicability in understanding and influencing health-related behaviors and intentions. In the context of CR, successful participation depends not only on patients’ physical readiness but also on their willingness to change, motivation, and commitment to achieving rehabilitation goals. Therefore, the TM provides a useful framework for understanding behavioral readiness among patients undergoing cardiac surgery, as rehabilitation outcomes are influenced by physical capacity, attitudes, emotional status, and decision-making processes. The TM has been applied in studies examining exercise behavior and participation in cardiac rehabilitation among cardiovascular populations [3,5,6].

Despite the importance of exercise readiness as a prerequisite for successful participation in comprehensive CR programs, this topic has received limited attention in the international literature. An important contribution to this field was the development of the Readiness to Change Exercise Questionnaire by Kheawwan et al.; however, evidence regarding its application among patients undergoing cardiac surgery remains scarce [5].

Huang et al. investigated exercise behavior and physical activity at 3 and 6 months following open-heart surgery using questionnaires based on the TM framework [6]. Their findings demonstrated that patients were able to maintain adequate levels of physical activity six months after surgery. These findings support the notion that behavioral readiness plays an important role in the adoption and maintenance of physical activity following cardiac surgery and highlight the potential value of stage-based approaches in cardiac rehabilitation.

Despite the growing recognition of the importance of behavioral readiness in cardiac rehabilitation, limited evidence is available regarding readiness for exercise among patients recovering from cardiac surgery, particularly in European populations. Therefore, further research is needed to identify factors associated with exercise readiness and to support the development of individualized rehabilitation interventions. The present study aimed to evaluate patients’ readiness to exercise following cardiac surgery and to identify the factors associated with exercise readiness.

## 2. Materials and Methods

### 2.1. Trial Design

A cross-sectional observational study was conducted. The dependent variable was readiness for exercise, while the independent variables included the demographic and clinical characteristics of the participants.

### 2.2. Participants

The study population consisted of patients who underwent cardiac surgery, including coronary artery bypass grafting (CABG), valve replacement, or a combination of procedures, at a general hospital in Athens, Greece. A total of 140 eligible patients underwent cardiac surgery during the study period. Readiness for exercise was assessed three months after surgery through a telephone interview.

**Figure 1:**
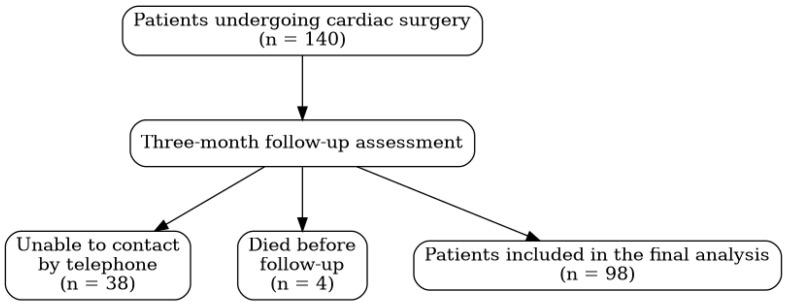
Flow chart of patient selection and follow-up.

Of the 140 eligible patients, 38 could not be contacted and were therefore unavailable for assessment. In addition, four patients had died before the three-month follow-up. Consequently, the final study sample comprised 98 patients who completed the assessment and were included in the analysis (Figure 1).

Data collection was conducted between April and August 2024.

Inclusion criteria:

- Patients who had undergone cardiac surgery (coronary artery bypass grafting, valve replacement, or a combination of procedures) within the previous three months.
- Age ≥ 18 years.
- Adequate knowledge of the Greek language.
- Absence of medical contraindications to physical activity.

Exclusion criteria:

- Incomplete clinical data in the medical record.
- Cognitive impairment or inability to provide informed responses during the telephone interview.

Patients who could not be contacted by telephone and patients who died before the three-month assessment were not excluded a priori but were unavailable for follow-up assessment and were therefore not included in the final analysis.

### 2.3. Sample

The sample size was determined by feasibility and the availability of eligible participants.

### 2.4. Procedure

The Greek version of the “Readiness to Change Exercise Questionnaire” was used to assess the readiness to exercise, developed by Kheawwan et al. in 2016 [5]. This tool assesses patients’ readiness to exercise after cardiac surgery. It consists of 13 questions (5-point Likert scale), divided into four subscales according to the TM. Specifically, 2 questions fall into the pre-contemplation stage, 4 into the contemplation stage, 5 into the preparation stage, and 2 into the action stage. To answer each question, there are 5 possible answers on an agreed scale (1 = strongly disagree to 5 = strongly agree). Participants were asked to select the response that best described their current readiness for exercise. To classify patients by degree of readiness for exercise, scores of all questions are collected at each subscale. Then, the sum of each subscale is divided by the number of questions in each subscale and an average value is obtained. The subscale with the highest mean value is that which characterizes the patient in terms of his/her willingness to exercise. Patients were classified into one of four stages according to the subscale with the highest mean score. For bivariate and logistic regression analyses, participants classified in the action stage were considered ready for exercise (“Yes”), whereas those classified in the pre-contemplation, contemplation, or preparation stages were considered not ready for exercise (“No”). This approach was adopted to distinguish patients who had already adopted regular exercise behavior from those who had not yet achieved this stage. No ties between subscale mean scores were observed.

The questionnaire was translated into Greek with the permission of the original authors using the forward–backward translation method. Initially, two independent bilingual translators translated the instrument from English into Greek. The resulting Greek version was subsequently provided to two additional bilingual translators, who were blinded to the original questionnaire and independently performed a back-translation from Greek into English.

The original English version and the back-translated version were then compared to identify and resolve any conceptual discrepancies between the items. During this process, linguistic and cultural differences were carefully considered to ensure conceptual equivalence and cultural appropriateness. Following the necessary revisions, the final Greek version of the questionnaire was established.

At the next stage, the questionnaire was pilot-tested in a sample of 30 individuals to evaluate its clarity and comprehensibility. The pilot study also provided preliminary evidence regarding the internal consistency of the Greek version of the instrument. These participants were not included in the final sample of the present study.

Also, a specially designed, structured questionnaire developed by the researchers was used to collect demographic and clinical characteristics of the sample.

The Greek version of the “Readiness to Change Exercise Questionnaire” was used to assess readiness to exercise via telephone interview 3 months after the surgery. The time required to complete the questionnaire was less than 10 min.

### 2.5. Ethical Consideration

This study was conducted according to the Declaration of Helsinki ethical principles [7]. The research protocol was approved by the Research Ethics Committee of the National and Kapodistrian University of Athens (Protocol No. 235/8 December 2017) and the Scientific/Ethics Committee of the general hospital where data collection took place (Protocol No. 342/21 December 2017).

### 2.6. Data Analysis

Categorical variables are presented as absolute (n) and relative (%) frequencies, whereas continuous variables are presented as means, standard deviations, and medians, as appropriate. The Kolmogorov–Smirnov test and visual inspection of normality plots were used to assess the distribution of continuous variables.

As previously described, the dependent variable was readiness for exercise, whereas the independent variables included the demographic and clinical characteristics of the sample. The association between a continuous variable and the binary outcome variable was assessed using the independent-samples *t*-test when the continuous variable followed a normal distribution and the Mann–Whitney U test when the assumption of normality was not met.

The chi-square test was used to assess associations between categorical variables, whereas the chi-square test for trend was applied to evaluate associations involving ordinal variables.

Variables with a significance level of *p* < 0.20 in the bivariate analysis were entered into a multivariable logistic regression model. A backward stepwise logistic regression procedure was used to identify independent predictors of readiness for exercise. Odds ratios (ORs), 95% confidence intervals (CIs), and *p*-values are reported for the final model.

The level of statistical significance was set at *p* < 0.05 (two-tailed). Statistical analyses were performed using SPSS version 21.0 Statistical analyses were performed using IBM SPSS Statistics for Windows, Version 21.0 (IBM Corp., Armonk, NY, USA).

## 3. Results

The sample included 98 patients. Table 1 presents the demographic characteristics of patients. The mean age (±standard deviation) of the patients was 64.2 (±10.7) years, while the majority were male (72.4%), of compulsory education (61.2%), married (67.3%), retired (55.1%), and lived with family/partner (75.5%).

Table 2 presents patients’ responses regarding their readiness for exercise. In the pilot study of 30 patients, the Cronbach’s alpha coefficient for the pre-contemplation subscale was 0.68, for “contemplation” was 0.64, for “preparation” was 0.81, and for “action” was 0.95. The Cronbach’s alpha coefficient in the main study was 0.79 for the “pre-contemplation” subscale, 0.6 for “contemplation”, 0.64 for “preparation”, and 0.95 for the “action” subscale. Overall, the internal consistency of the questionnaire ranged from acceptable to excellent across subscales, although the contemplation and preparation subscales demonstrated relatively modest Cronbach’s alpha values, indicating that further psychometric evaluation of the Greek version is warranted. A total of 53.1% of patients had a higher mean score on the “action” subscale, 24.5% on preparation, 11.2% on contemplation, and 11.2% on pre-contemplation.

Table 3 presents the bivariate correlations between the independent variables of our study and readiness for action. Variables associated with readiness for exercise at a significance level of *p* < 0.20 in the bivariate analysis were entered into a multivariable logistic regression model. For this reason, multivariate logistic regression was applied, the results of which are presented in Table 4. According to the multivariable logistic regression analysis, male sex and divorced/widowed marital status were independently associated with higher odds of being classified as ready for exercise compared with female sex and unmarried status, respectively. In addition, higher levels of preoperative physical activity were associated with greater readiness for exercise. The final model explained approximately 25% of the variance in readiness for exercise (Nagelkerke R^2^ = 0.25).

A total of 98 patients were included in the analysis, with 51.3% being classified in the action stage. Variables associated with readiness for exercise at *p* < 0.20 in the bivariate analysis were considered for inclusion in the multivariable logistic regression model. The final model included four parameters, resulting in an adequate events-per-variable ratio and minimizing the risk of model overfitting.

The overall model was statistically significant (Omnibus test, *p* = 0.023). The Hosmer–Lemeshow goodness-of-fit test indicated an adequate fit of the model to the data (χ^2^ = 5.84, *p* = 0.67). The model explained 25.3% of the variance in readiness for exercise according to the Nagelkerke R^2^ and correctly classified 75.6% of cases. These findings support the adequacy and stability of the final logistic regression model.

## 4. Discussion

The present cross-sectional observational study aimed to evaluate patients’ readiness to exercise following cardiac surgery, including CABG, valve replacement, or a combination of procedures. Patients’ readiness to exercise was assessed using the Readiness to Change Exercise Questionnaire developed by Kheawwan et al. [5]. To the best of our knowledge, this is the first study to apply this instrument in the Greek population. A total of 98 patients participated in the study.

The findings demonstrated that sex, marital status, and preoperative physical activity levels were significantly associated with readiness for postoperative exercise. Specifically, male patients, divorced or widowed individuals, and patients with moderate or high levels of preoperative physical activity were more likely to be classified as ready for exercise than female patients, unmarried participants, and those with low levels of preoperative physical activity.

A review of both the Greek and international literature revealed a paucity of studies examining exercise readiness among patients undergoing cardiac surgery. To our knowledge, no previous study in Greece has investigated this topic, highlighting both the novelty of the present research and the need for further studies in this field.

None of the participants in the present study had previously enrolled in a formal CR program. Instead, all patients received routine postoperative care, including oral and written instructions regarding disease management and recovery. Notably, more than half of the participants (53.1%) were classified in the “action” stage of the Transtheoretical Model, indicating a relatively high level of readiness to engage in exercise following surgery.

Similar findings were reported by Huang et al. [6], who observed that the largest proportion of patients were classified in the “action” stage six months after open-heart surgery. In their study, the “maintenance” stage represented the second-most frequent category. The maintenance stage was not evaluated in the present study because of the shorter follow-up period. The relatively high proportion of participants classified in the action stage may reflect a combination of favorable clinical and behavioral characteristics of the study sample, including a relatively high prevalence of moderate-to-high preoperative physical activity and a low incidence of major postoperative complications and rehospitalizations.

The findings may also support the importance of the Transtheoretical Model as a conceptual framework for understanding exercise behavior after cardiac surgery. According to the model, individuals in the action stage have already initiated behavioral change and are more likely to maintain health-promoting behaviors when appropriate support is provided. Therefore, assessing readiness for exercise may assist clinicians in tailoring interventions according to each patient’s stage of change and facilitate more individualized rehabilitation planning.

The present findings should also be interpreted within the broader context of contemporary cardiac rehabilitation research. Recent international guidelines and position statements emphasize that exercise training remains a cornerstone of cardiac rehabilitation and is associated with improved functional capacity, quality of life, and long-term cardiovascular outcomes among patients recovering from cardiac surgery and other cardiovascular conditions. Furthermore, current rehabilitation models increasingly recognize the importance of behavioral, psychosocial, and motivational factors that influence patients’ engagement in physical activity and adherence to rehabilitation programs. In this context, assessing patients’ readiness for exercise may provide valuable information to identify individuals who require additional support and to tailor rehabilitation strategies according to patients’ needs and stage of behavioral change [4,8,9].

An important finding of the present study was the association between male sex and greater readiness for exercise. Previous research has shown that women, particularly those older than 65 years, are less likely to engage in physical activity and participate in CR programs than men [10]. Furthermore, men represent the majority of participants enrolled in CR programs worldwide [11,12,13]. This observation may partly reflect the higher prevalence of cardiovascular disease among men, which is consistent with the demographic characteristics of our sample, where 72.4% of participants were male [14]. However, Huang et al. [6] did not identify a significant association between sex and exercise readiness, suggesting that additional factors may influence exercise behavior following cardiac surgery.

Divorced or widowed participants demonstrated greater readiness for exercise than unmarried individuals. Previous studies have reported that marital status may influence adherence to cardiac rehabilitation programs, with individuals living alone being more likely to discontinue rehabilitation [15]. Although this finding appears inconsistent with our results, it should be interpreted with caution. The relatively small number of divorced or widowed participants and the wide confidence interval of the estimated odds ratio suggest limited precision and possible instability of the estimate. Moreover, marital status should not be considered a direct indicator of social support, as many divorced or widowed individuals may still benefit from strong family and community networks. Therefore, this finding should be considered exploratory and warrants confirmation in larger studies.

The positive association between preoperative physical activity and postoperative exercise readiness is consistent with previous evidence. Van Laar et al. [16] reported that reduced preoperative physical activity was associated with an increased risk of postoperative complications. Individuals who maintain an active lifestyle before surgery are likely to have better physical conditioning and may therefore be more willing and able to participate in exercise during the recovery period.

The relationship between age and participation in CR has also been examined in previous studies. Socha et al. and Jegier et al. reported that age may influence participation and outcomes in rehabilitation programs [8,17]. However, neither Van Laar et al. [16] nor the present study identified a statistically significant association between age and readiness for exercise. This finding may be attributable to the relatively limited sample size.

Most participants in the present study underwent CABG, followed by valve replacement surgery. This finding is consistent with European epidemiological data indicating that CABG remains one of the most frequently performed cardiac surgical procedures [18]. Nevertheless, the type of surgery was not significantly associated with readiness for exercise in our sample.

Despite its contributions, the present study has several limitations. To the best of our knowledge, this is the first study conducted in Greece to evaluate patients’ readiness to exercise following cardiac surgery using the Readiness to Change Exercise Questionnaire. In addition, the study addresses an underexplored area of cardiac rehabilitation by examining behavioral readiness for exercise. This factor may significantly influence patients’ participation in rehabilitation programs and postoperative recovery.

However, several limitations should be acknowledged. First, the study was conducted in a single cardiac surgery center, which may limit the generalizability of the findings to other healthcare settings and populations. Second, the relatively small sample size may have reduced the statistical power to detect associations between certain demographic or clinical variables and readiness for exercise. Furthermore, no a priori sample size or power calculation was performed. Third, data were collected through telephone interviews, introducing the possibility of recall bias and socially desirable responses. Furthermore, the cross-sectional design does not allow causal inferences regarding the relationships identified between patient characteristics and exercise readiness. In addition, readiness for exercise was assessed only once, three months after surgery, and no baseline assessment was available; therefore, changes in readiness over time could not be evaluated. Some regression estimates, particularly those related to marital status, were accompanied by wide confidence intervals, suggesting limited precision and possible instability of these findings. Moreover, social support was not directly measured, although it may be an important determinant of exercise readiness and could partly explain the observed association between marital status and readiness for exercise.

Another limitation of the present study is the relatively high participant attrition rate during follow-up. Approximately 30% of the initially eligible patients were not included in the final analysis because they could not be contacted during follow-up or had died before the three-month assessment. Consequently, selection bias cannot be excluded, and the final study sample may not be fully representative of the overall population of patients undergoing cardiac surgery. Patients who completed the study may have differed from those who were not included in terms of motivation, health status, or willingness to engage in postoperative exercise. Therefore, the findings should be interpreted with caution, and future multicenter studies with larger and more representative samples are warranted.

Although the Greek version of the Readiness to Change Exercise Questionnaire demonstrated acceptable to excellent internal consistency across most subscales, the contemplation and preparation subscales exhibited relatively modest Cronbach’s alpha values. Furthermore, the psychometric evaluation performed in the present study was limited to internal consistency assessment. Therefore, additional studies involving larger samples and more comprehensive psychometric analyses are needed to provide further evidence regarding the reliability and validity of the Greek version of the instrument.

Future multicenter studies with larger samples and longitudinal follow-up are warranted to further investigate the determinants of exercise readiness and their impact on postoperative recovery and long-term rehabilitation outcomes.

## 5. Conclusions

Sex, marital status, and preoperative level of physical activity were significantly associated with readiness for postoperative exercise in patients undergoing cardiac surgery. These findings may help nurses identify patients who require additional support to engage in postoperative exercise and tailor interventions according to individual needs, thereby facilitating postoperative recovery. Further research, based on a larger sample of patients, multicenter studies, and interventions that could improve patients’ readiness for postoperative exercise, is needed.

## Figures and Tables

**Table 1:** Demographic characteristics of patients.

Characteristic	Ν	%
Sex		
Female	27	27.6
Male	71	72.4
Educational status		
Low (compulsory education)	60	61.2
Medium (secondary education)	28	28.6
High (university)	10	10.2
Marital status		
Married	66	67.3
Divorced/widower	16	16.3
Not married	16	16.3
Living conditions		
With a family/another person	74	75.5
Alone	24	24.5
Employment		
Employed	33	33.7
Unemployed	6	6.1
Retired	54	55.1
House holding	5	5.1
	Mean	±SD
Age	64.2	10.7

SD: standard deviation.

**Table 2:** Descriptive results for the four stages of the Patient Readiness for Exercise Questionnaire.

Score	Mean	Standard Deviation	Median	Minimum	Maximum	N	(%)
Pre-contemplation	2.0	1.2	1.3	1	5	11	11.2
Contemplation	2.4	1.0	2.5	1	4.5	11	11.2
Preparation	3.6	0.9	3.7	1.2	5	24	24.5
Action	3.4	1.6	4	1	5	52	53.1

**Table 3:** Variable correlations between independent variables and readiness for action.

Characteristic	Readiness for Action		Statistical Test	*p*-Value
	No	Yes		
Biological gender			Chi-Square	0.1
Female	16 (59.3)	11 (40.7)		
Male	30 (42.3)	41 (57.7)		
Age ^b^	65.2 (11.2)	63.4 (10.3)	*t*-Test	0.4
Educational status			Chi-Square Trend Test	0.5
Low (compulsory education)	30 (50.0)	30 (50.0)		
Medium (secondary education)	12 (42.9)	16 (57.1)		
High (university)	4 (40.0)	6 (60.0)		
Marital status			Chi-Square	0.1
Married	34 (51.5)	32 (48.5)		
Divorced/widower	4 (25.0)	12 (75.0)		
Not married	8 (50.0)	8 (50.0)		
Living conditions			Chi-Square	0.05
With family/another person	39 (52.7)	35 (47.3)		
Alone	7 (29.2)	17 (70.8)		
Employment			Chi-Square	0.3
Employed	18 (54.5)	15 (45.5)		
Not in paid employment (unemployed, retired, householding)	28 (43.1)	37 (56.9)		
Surgery			Chi-Square	0.4
Coronary artery bypass	24 (41.4)	34 (58.6)		
Valve replacement	14 (58.3)	10 (41.7)		
Combination	6 (54.5)	5 (45.5)		
Other surgery	2 (40.0)	3 (60.0)		
Urgency			Chi-Square	0.1
Emergent surgery	19 (57.6)	14 (42.4)		
Programmed surgery	27 (41.5)	38 (58.5)		
Critical preoperative state			Chi-Square	0.9
No	41 (47.1)	46 (52.9)		
Yes	5 (45.5)	6 (54.5)		
Previous cardiac surgery			Chi-Square	0.4
No	43 (45.7)	51 (54.3)		
Yes	3 (75.0)	1 (25.0)		
Surgery duration (minutes) ^b^	189.0 (42.1)	184.7 (43.6)	*t*-Test	0.6
The period of extracorporeal circulation during surgery (minutes) ^b^	98.7 (33.3)	96.3 (30.7)	*t*-Test	0.7
Plasma creatinine (preoperatively) ^e^	0.98 (1.8)	1.08 (6.2)	Mann–Whitney Test	0.9
Left ventricular ejection fraction ^b^	52.5 (11.8)	53.5 (10.8)	*t*-Test	0.8
Days of hospitalization postoperatively ^c^	8.6 (5.1)	7.4 (1.6)	*t*-Test	0.1
Postoperative complications			Chi-Square	0.8
No	39 (46.4)	45 (53.6)		
Yes	7 (50.0)	7 (50.0)		
Mobility problems			Chi-Square	0.03
No	38 (43.2)	50 (56.8)		
Yes	8 (80.0)	2 (20.0)		
Level of physical activity preoperatively			Chi-Square Trend Test	0.001
Low	17 (77.3)	5 (22.7)		
Moderate	15 (45.5)	18 (54.5)		
High	14 (32.6)	29 (67.4)		
Comorbidity			Chi-Square	0.8
No	9 (42.9)	12 (57.1)		
Yes	37 (48.1)	40 (51.9)		
BMΙ (kgr/m^2^) ^b^	27.9 (5.2)	26.4 (3.2)	*t*-Test	0.08
Tobacco smoking			Chi-Square Trend Test	0.7
Never	19 (45.2)	23 (54.8)		
Present use	5 (41.7)	7 (58.3)		
Past use and interruption	22 (50.0)	22 (50.0)		
Alcohol consumption (daily use)			Chi-Square Trend Test	0.1
Never	37 (51.4)	35 (48.6)		
Present use	3 (42.9)	4 (57.1)		
Past use and interruption	6 (31.6)	13 (68.4)		

Values are expressed as n (%) unless stated otherwise. ^b^: mean (SD), ^c^: *t*-test, ^e^: median (range).

**Table 4:** Multivariate logistic regression with the dependent variable as the readiness to exercise.

Characteristic	Odds Ratio	95% CI for the Odds Ratio	*p*-Value
Male to female	3.3	1.0–10.4	0.042
Divorced/widowers to nonmarried	9.7	1.5–64.2	0.018
Preoperative physical activity: Moderate vs. Low	5.2	1.4–18.7	0.012
Preoperative physical activity: High vs. Low	7.4	2.1–26.0	0.002

CI: confidence interval.

## Data Availability

The data presented in this study are available on reasonable request from the corresponding author. The data are not publicly available due to privacy and ethical restrictions related to human participant data.

## Abbreviations

The following abbreviations are used in this manuscript:

TM	Transtheoretical Model
CR	Cardiac Rehabilitation
CABG	Coronary Artery Bypass Graft
